# Enhanced anti-tumor efficacy of S3I-201 in breast cancer mouse model through Wharton jelly- exosome

**DOI:** 10.1186/s12935-024-03501-3

**Published:** 2024-09-18

**Authors:** Masoomeh Hosseini, Rana Ezzeddini, Seyed Mahmoud Hashemi, Sara Soudi, Amir Salek Farrokhi

**Affiliations:** 1 Department of Immunology, Pasteur Institute of Iran, P.O. Box: 1316943551, Tehran, Iran; 2 Department of Immunology, Semnan University of Medical Sciences, Semnan, Iran; 3 Department of Clinical Biochemistry, Tarbiat Modares University, P.O. Box: 156352698, Tehran, Iran; 4 Department of Immunology, School of Medicine, Shahid Beheshti University of Medical Sciences, Tehran, Iran; 5 Department of Immunology, Tarbiat Modares University, Tehran, Iran

**Keywords:** Breast cancer, STAT3 inhibitor, WJ-mesenchymal stem cells, Exosome, Drug delivery

## Abstract

**Objective:**

Exosomes, membrane-enveloped vesicles found in various cell types, including Wharton’s jelly mesenchymal stem cells, play a crucial role in intercellular communication and regulation. Their use as a cell-free nanotechnology and drug delivery system has attracted attention. Triple-negative breast cancer (TNBC) is a major global health problem and is characterized by a high mortality rate. This study investigates the potential of Wharton’s Jelly mesenchymal stem cell-derived exosomes (WJ-Exo) as carriers of S3I-201 and their effects on STAT3 expression in breast cancer cell lines, and evaluates whether these exosomes can enhance the anti-tumor effect of S3I-201.

**Methods:**

The filtered WJ-Exos were analyzed by Transmission Electron Microscopy (TEM), Scanning electron microscopy (SEM), Dynamic Light Scattering (DLS), flow cytometry, and Western blotting. These exosomes were then used for loading with S3I-201, resulting in the nano-formulation WJ-Exo(S3I-201). The effect of WJ-Exo(S3I-201) on 4T1 cancer cells was investigated in vitro using MTT assay, flow cytometry, wound healing assay, Western blotting and Quantitative Real-Time Polymerase chain reaction (qPCR) analysis. Finally, the therapeutic efficacy of the nano-formulation was investigated in vivo using a tumor-bearing mouse model.

**Results:**

In vitro experiments showed that co-incubation of 4T1 cells with the nano-formulation resulted in a significant reduction in p-STAT3 levels, induction of apoptosis, modulation of Bcl-2, Bax and caspase-3 protein and gene expression, and inhibition of migration. In vivo, treatment of tumor-bearing mice with WJ-Exo(S3I-201) showed a strong antitumor effect that exceeded the efficacy observed in the S3I-201 group.

**Conclusion:**

Our results demonstrate that WJ-Exo is an effective carrier for targeting S3I-201 to tumor cells and enhances the therapeutic efficacy of S3I-201 in tumor-bearing mice.

## Introduction

Triple-negative breast cancer (TNBC) is a highly aggressive and lethal subtype of breast cancer that contributes significantly to global mortality and morbidity [1]. Recent efforts to identify targeted molecules for the treatment of TNBC via genomic profiling have uncovered critical alterations, particularly the overexpression and abnormal activation of Signal Transducer and Activator of Transcription 3 (STAT3) [2, 3], which plays a central role in controlling vital cellular processes. These processes include cell survival, proliferation, cell cycle progression, anti-apoptosis, migration, invasion, angiogenesis, chemo-resistance, immunosuppression and stem cell self-renewal and differentiation, with STAT3 regulating the expression of genes associated with these functions [4]. S3I-201, a small molecule inhibitor targeting STAT3, has shown potent anticancer activity in in vitro and in vivo models of TNBC [5–7]. Despite the development of various cancer therapies such as chemotherapy, surgery, radiotherapy and immunotherapy, their potential to damage healthy cells and cause systemic side effects makes it necessary to explore new, selective approaches to target and eliminate cancer cells.

Exosomes are among the emerging strategies that have attracted considerable scientific attention [8–10]. They are a promising option for delivery due to their inherent properties that ensure safe and efficient transportation of cargo [11]. Compared to micelles, liposomes, and polymeric nanoparticles, exosomes serve as a natural delivery system capable of evading phagocytosis, prolonging half-life in blood, and exhibiting optimal biocompatibility, minimizing potential long-term safety concerns [12–14]. Natural, unmodified exosomes have been shown in various studies to possess inherent tumor-targeting properties [15, 16]. In addition, studies have confirmed that exosomes derived from the patient’s own cells have lower immunogenicity [17, 18]. In contrast, artificial delivery systems inevitably present challenges in terms of immunogenicity and toxicity [19, 20]. The increasing interest in the development of exosomes, driven by the growing demand in the health sector, is remarkable. Exosomes, natural particles on the nanoscale of 30 to 150 nm, offer significant advantages over artificially produced nanoparticles. Over the past decade, basic research and clinical studies focused on exosomes have gained momentum in various medical fields [21–23]. Given the myriad functions performed by the different types of exosomes secreted by almost all cells, selecting the optimal cells for research is a critical consideration.

Mesenchymal stem cells (MSCs), which play an important role in stem cell research in regenerative medicine, cell therapy and tissue engineering, are promising candidates. Research on exosomes of MSCs (MSC exosomes) is very valuable as it combines the advantages of exosomes with the properties of MSCs. Numerous studies highlight the therapeutic potential of MSC exosomes in various diseases, including tumors [24], neurodegenerative diseases [25], cardiovascular [26] and cerebrovascular diseases [27], and wound repair [28]. Therefore, MSC exosomes can be considered as potential nano-therapeutics. In addition, MSC exosomes as natural nano-drug carriers show promising results in current research for the treatment of diseases [29, 30].

The human umbilical cord (UC) is a non-invasive and abundant source of MSCs that has attracted attention due to its safety for ethical concerns. Stem cells are found in various organs and tissues, have regenerative potential and play a key role in tissue repair throughout life. Adult stem cells (ASCs), including MSCs and hematopoietic stem cells, are isolated from sources such as bone marrow, adipose tissue and dental pulp. However, their limited number and invasive collection pose a challenge [31]. To address this problem, stem cells from perinatal sources such as Wharton’s jelly from the umbilical cord have been explored. These cells are more primitive and readily available. They are obtained non-invasively at birth and show only minimal changes due to aging and environmental stress [32, 33].

The aim of this study was to develop a nano-carrier system using exosomes from Wharton’s Jelly (WJ-Exo) mesenchymal stem cells for the encapsulation of S3I-201. The studies were performed on the 4T1 breast cancer cell line and an in vivo TNBC model. Exosomes were isolated from WJ-MSCs media by filtration and their potential as carriers for the anticancer drug S3I-201 was investigated. WJ exosomes enriched with S3I-201 (WJ-Exo(S3I-201)) showed enhanced anti-tumor activity compared to the WJ-Exo and S3I-201 groups. Our results indicate that WJ-Exo(S3I-201) enhances the anti-tumor efficacy of chemotherapy by suppressing STAT3 expression and down-regulating tumor growth by restoring inflammatory status in tumor-bearing mouse model.

## Experimental and materials

### General design of the study

This study aimed to investigate the potential of using WJ-Exo as carriers of the anticancer drug S3I-201 in treating TNBC. The research encompassed several key stages: characterization of the WJ-Exos through various analyses including TEM, SEM, DLS, flow cytometry, and Western blotting; loading of the exosomes with S3I-201 to create the nano-formulation WJ-Exo(S3I-201); assessment of the effects of WJ-Exo(S3I-201) on 4T1 cancer cells in vitro using a range of techniques such as MTT assay, flow cytometry, wound healing assay, Western blotting, and qPCR; and evaluation of the therapeutic efficacy of the nano-formulation in vivo using a tumor-bearing mouse model.

### Reagents

RPMI-1640 medium (Gibco, NY, USA), Dulbecco’s Modified Eagle’s Medium F12 (DMEM F12) (Gibco, Grand Island, NY), fetal bovine serum (FBS) (Gibco, Grand Island, NY), anti-human antibodies labeled with fluorochromes against CD90, CD105, CD45, CD73, and CD14 (all from eBioscience), a Bicinchoninic Acid (BCA) kit (Thermo Fisher Scientific, USA), Exosome extraction kit (EXOCIB; Cibbiotech, Iran), Anti-CD63 (sc-5275, 1:200), anti-CD9 (sc-13118, 1:200), anti-CD81 (sc-166029, 1:100), and anti-β-actin (sc-47778, 1:300) (Santa Cruz Biotechnology, Dallas, Texas, USA). FITC-conjugated mouse anti-human CD63, APC-conjugated mouse anti-human CD9, and PE-conjugated mouse anti-human CD81 (BD, USA), S3I-201 (Sigma-Aldrich), MTT solution (5 mg/mL) (Sigma-Aldrich, USA), Annexin V‑PE/7‑aminoactinomycin D (7‑AAD) apoptosis detection kit (BD Biosciences, San Diego, CA, USA), specific antibodies (P-STAT3, STAT3, cleaved caspase-3, Bcl-2, and β-actin at 1:1000 dilution, Cell Signaling Technology, Danvers, MA, USA) overnight at 4 °C. Following the incubation with an HRP-conjugated anti-rabbit secondary antibody (1:5000, Cell Signaling Technology, Danvers, MA, USA), Trizol reagent (Roche Diagnostics GmbH, Mannheim, Germany), cDNA synthesis kit (Takara Biotechnology, Otsu, Shiga, Japan), SYBR-Green Master Mix (Ampliqon, Denmark).

### Human WJ-MSC isolation

Anonymous umbilical cord units (15–20 cm) were obtained from postpartum women undergoing normal labor at Royan Institute (Tehran, Iran), focusing on individuals aged 20–40 years who delivered healthy infants (> 38 weeks). Informed consent was obtained from the tissue donors before using the samples. Under sterile conditions, UC units were freshly collected, cleaned with cold saline and antibiotics, and blood vessels were extracted to obtain Wharton’s Jelly [34] tissue. Subsequently, the WJ was mechanically cut into 1 cc pieces and evenly transferred into 75 cm² T-flasks (SPL Life Sciences, South Korea) filled with Dulbecco’s Modified Eagle’s Medium F12 (DMEM F12) (Gibco, Grand Island, NY) supplemented with 15% fetal bovine serum (FBS) (Gibco, Grand Island, NY). Cultivation took place in a standard environment at 37 °C, 21% O₂ and 5% CO₂. After approximately one week, the culture medium was refreshed. After reaching decellularized WJ, the explants were removed and the cells were passaged at 80% confluence, showing fibroblast-like morphology. Routine observation, analysis and documentation of the MSCs at the morphological level were performed using an inverted light microscope (Nikon, Japan).

#### Characterization of human WJ-MSC

In accordance with the minimum criteria established by the International Society for Cell & Gene Therapy (ISCT) for the definition of human MSCs, the cells obtained were characterized. These criteria include plastic adherence, the expression of CD73, CD90 and CD105, the absence of hematopoietic and endothelial markers such as CD14, CD34 and CD45, and the ability to differentiate in vitro into adipocyte and chondrocyte lines [35].

#### Assessment of WJ-MSC adherence and surface antigen expression

The adherence of WJ-MSCs to standard culture plates and their morphological characteristics were examined using an inverted light microscope (Nikon, ECLIPSE TS100, Japan) with phase-contrast optics. The expression of specific surface antigens on WJ-MSCs was analyzed using a FACSCalibur flow cytometer (BD Biosciences, San Jose, CA, USA). Cells were detached with trypsin-EDTA (0.05%) solution (Gibco, NY, USA) and WJ-MSCs were treated with anti-human antibodies labeled with fluorochromes targeting specific markers such as CD90, CD105, CD45, CD73, and CD14 (all from eBioscience) at 4 °C for 30 min in the absence of light. Isotype antibodies served as controls to exclude nonspecific binding, and FlowJo software (San Jose, CA) was used to analyze results.

#### Confirmation of multilineage potential of WJ-MSCs

The multilineage potential of the WJ-MSCs was confirmed by exposing them to adipogenic and chondrogenic induction media, staining with Oil Red-O and Alizarin Red-S (Sigma-Aldrich, Missouri, USA), respectively. 3 × 10^4^ third passage MSCs were cultured in each well of 4-well plates under standard conditions (21% O2, 5% CO2, 37 °C, humidified incubator) for 20 days. Subsequently, the induction media were replaced by wells washed with phosphate buffered saline (PBS), fixed and stained. Cells that had undergone differentiation were examined using a reversed light microscope (Nikon, Japan) with phase contrast optics.

### Preparation of WJ-Exo

WJ-MSCs-conditioning medium (WJ-MSCs-CM) was obtained from cells in passages 2 or 3. First, WJ-MSCs were cultured in 150 cm2 T-flasks (SPL Life Sciences, South Korea) under standard conditions (5% CO^2^, 37 °C). After reaching 70–75% confluence, the medium was gradually changed to a lower FBS content every 2 days and finally switched to FBS-free conditions. Cells were then cultured in serum-free medium for three days and the resulting CM was collected and filtered through a 0.22 μm membrane filter to remove debris.

Exosomes were isolated according to the manufacturer’s protocol (EXOCIB; Cibbiotech, Iran). In summary, the serum-free culture medium was centrifuged at 3000 rpm for 10 min at room temperature to remove debris. Reagent A was added to WJ-MSCs-CM at a ratio of 1:5, mixed by vortexing for 5 min and incubated overnight at 4 °C. After centrifugation at 3000 rpm for 40 min at 4 °C, the supernatant was discarded and the exosomes were resuspended in reagent B and then stored at -80 °C for further experiments.

#### Exosome protein quantification

A bicinchoninic acid (BCA) kit (DNAbiotech Co.) was used to quantify exosomal protein content. Bovine serum albumin (BSA) standards were serially diluted to prepare eight concentrations. Then, 25 µl of the BSA standards or samples were combined together with 75 µl of the BCA working reagent in a 96-well ELISA plate and mixed thoroughly. After incubating the plate at 60 °C for 60 min, the optical density (OD) was measured at 560 nm using an ELISA reader system (BioTek, USA).

#### WJ-Exosome characterization

WJ exosomes were characterized according to the MISEV2018 guidelines. This included morphological assessment (spheroid shape), sizing (30–150 nm) and expression of surface markers (CD9, CD63, CD81). Size and quality of the purified exosomes were assessed by transmission electron microscopy (TEM), scanning electron microscopy (SEM) and dynamic light scattering (DLS). For TEM, exosomes were fixed in 1% glutaraldehyde (Sigma), placed on carbon-coated grids and examined with a LEO 906 transmission electron microscope (Carl Zeiss, Germany). The grids were prepared for TEM by washing with PBS, staining with 1% uranyl acetate and imaging with an Orius 200 camera with Digital Micrograph software (Gatan Inc, USA) at 80 kV.

Glutaraldehyde-fixed exosomes on glass slides were used for SEM analysis, which were observed with a TESCAN MIRA3 FEG-SEM after gold–palladium sputtering at 15 kV.

DLS was used to determine the size distribution of the purified exosomes after dilution in PBS (1:6 ratio) and then examined using the Zetasizer (Malvern, UK). The resulting data were analyzed with the Malvern software (Zetasizer Ver. 7.11).

The expression of surface markers (CD63, CD9 and CD81) was analyzed by Western blotting and flow cytometry using antibodies from Santa Cruz Biotechnology and FITC/APC/PE-conjugated antibodies from BD, respectively. For flow cytometry, markers were detected using the FACSCalibur flow cytometer and analyzed using FlowJo software (San Jose, CA).

For Western blotting, exosomes were heated to 95 °C for 5 min in a PMSF-containing lysis buffer [36] and resolved by 12% sodium dodecyl sulfate-polyacrylamide gel electrophoresis (SDS-PAGE). They were then blotted onto nitrocellulose membranes and blocked with non-fat milk powder in Tris-buffered saline with Tween 20 (TBST) for 1 h. The membranes were then probed overnight at 4 °C with primary antibodies. After washing with TBST, membranes were treated with horseradish peroxidase (HRP)-conjugated secondary anti-rabbit or anti-mouse antibodies for 1 h. Protein bands were detected using X-ray films and enhanced chemiluminescence (ECL) reagents.

#### Loading of S3I-201 into WJ-Exo

To load therapeutic cargo into WJ-Exo, the anticancer agent S3I-201 (Sigma-Aldrich) was incorporated into WJ-Exo. Purified exosome samples (100 µL) were suspended in 200 µL of electroporation buffer (1.15 mM potassium phosphate, pH 7.2, 25 mM potassium chloride and 21% vol/vol OptiPrep). Different concentrations of S3I-201 (10, 100, 200, 300, 400, 500 µM) were combined with the exosome suspension at 4 °C and transferred to sterile electroporation cuvettes. Loading of the exosomes with S3I-201 was performed by applying a voltage of 600 V for 5 ms in 0.4 cm cuvettes using a multiporator (Eppendorf, USA). The mixture was then incubated at 37 °C for 30 min to facilitate recovery of the exosome membranes. The exosomes loaded with S3I-201 (WJ-Exo(S3I-201)) were isolated by centrifugation at 90,000 rpm for 1 h, and the loading efficiency of S3I-201 in exosomes was measured by HPLC-UV.

#### Determination of the S3I-201 load in exosomes

To determine S3I-201 loading in exosomes, drug loading in exosome studies was evaluated by HPLC. A standard curve for S3I-201 was first established using stock solutions of 10 mM S3I-201 in dimethyl sulfoxide (DMSO). S3I-201 standards from 1 to 500 µM (1, 10, 100, 200, 300, 400 and 500) were prepared with acetonitrile (MeCN). A C18 column (C18; 5 μm; 4.6 × 250 mm) (XBridge, Germany) with a mobile phase of H2O: acetonitrile (45:55, v/v) at 1 mL/min and 30 °C was used for the analysis and the absorbance was measured at 268 nm. The standard curve was constructed based on the area under the curve of the standards. For quantification of exosome active compounds, exosomes loaded with S3I-201 were combined with methanol (Merk, Germany), centrifuged at 13,000 rpm for 20 min at 4 °C, and the supernatant was evaporated with methanol. The solvent containing the exosomes loaded with S3I-201 was evaporated using a heating block (WiseTherm, Switzerland). The sample was then equilibrated with acetonitrile (Merk, Germany), sonicated for 25 min and injected (10 µL aliquots) into the HPLC system (Agilent 1200, USA).

### Cell culture

Mouse breast cancer cells, 4T1 (NCBI code: C604, ATCC number: CRL-2539), were purchased from the Pasteur Institute in Tehran, Iran. These cells were cultured in RPMI-1640 medium (Gibco, NY, USA) supplemented with 10% fetal bovine serum and 1% penicillin-streptomycin (Gibco, NY, USA) and maintained at 37 °C in a 5% CO2 environment.

### MTT assay

The cytotoxic activity of free S3I-201 and WJ-Exo(S3I-201) against 4T1 cells was determined using the standard 3-(4,5-dimethyl-2-thiazolyl)-2,5-diphenyl-2-H-tetrazolium bromide (MTT) assay. First, 4T1 cells (1 × 10^4^ cells/well) were cultured in a 96-well plate with complete media (RPMI-1640, 10% FBS and 1% antibiotic) for 24 h. Subsequently, 1 µg exosome with different concentrations of WJ-Exo (S3I-201) and free S3I-201 (10, 100, 200, 300, 400, 500 µM) were added to cell culture. After 48 h, MTT solution (5 mg/mL) (Sigma-Aldrich, USA) was added for 4 hours, followed by the addition of DMSO (Sigma-Aldrich, USA) to dissolve the formazan precipitate. Absorbance at 570 nm was measured using a spectrophotometer (Anthos, Austria) and cytotoxicity rates were determined in comparison to the negative control (DMSO only). All experiments were performed in triplicate and the IC50 values for loaded exosomes and free S3I-201 were calculated and compared using GraphPad Prism software.

### Investigation of S3I-201cytotoxicity on PBMCs

Peripheral blood mononuclear cells (PBMCs) were isolated from 7.5 ml of human whole blood by density centrifugation with a Ficoll gradient (Sigma-Aldrich, USA) at 850 g for 20 min at 20 °C, separating lymphocytes, monocytes and plasma. The layer containing the PBMCs was carefully removed from the original tube and transferred to a new 15 ml conical tube. The PBMCs were washed twice with 1× PBS containing 2% FBS, with each wash lasting 1 min. PBMCs were seeded at a density of 1 × 105 cells/well in 96-well plates with complete media (RPMI-1640, 10% FBS and 1% antibiotic) and treated with different concentrations of free S3I-201 (10, 100, 200, 300, 400, 500 µM). After a 48-hour incubation period, 20 µL of a 5 mg/mL MTT solution was added to each well, followed by 4-hour incubation at 37 °C. Optical density of samples evaluated by spectrophotometric analysis at 570 nm.

### Apoptosis assay

Phosphatidylserine exposure on the plasma membranes of cells, indicative of apoptosis in 4T1 cells, was detected using the Annexin VPE/7aminoactinomycin D (7AAD) apoptosis detection kit (BD Biosciences, USA) according to the manufacturer’s instructions. 4T1 cells (5 × 10^5^/well) were cultured in a 24-well plate with complete media for 24 h. Next, 5 µg WJ-Exo (S3I-201) loaded with 301.4 µM of S3I-201 (IC50 value) and 337.1 µM of free S3I-201 (IC50 value) were added to cell culture. This assay distinguishes between intact (Annexin V/7AAD), early apoptotic (Annexin V+/7AAD), late apoptotic (Annexin V+/7AAD+) and necrotic cells (Annexin V/7AAD+). In brief, cells were harvested after the treatment period with 0.25% trypsin, washed twice with cold PBS and resuspended in 100 µl 1X binding buffer. Addition of 5 µl Annexin VPE and 5 µl 7AAD followed by 15 min incubation at room temperature in the dark for 1 million cells. Subsequently, 400 µl of 1X binding buffer was added and flow cytometric analysis was performed using FlowJo software (Ashland, USA). Experiments were performed in triplicate.

### Western blot analysis

4T1 cells (7 × 10^5^ cells/well) were seeded in a six-well plate and allowed to grow to full confluence. Treatment medium consisting of free S3I-201 (337.1 µM), WJ-Exo(S3I-201) (5 µg exosome loaded with 301.4 µM of S3I-201), WJ-Exo or DMSO was then added to the cells. Protein extraction from 4T1 cells was performed using a cell lysis buffer (Cell Signaling Technology, USA). Protein concentration was determined using the BCA Protein Assay Kit (Thermo Fisher Scientific, USA) according to the manufacturer’s instructions. Equal amounts of protein were separated on 10% SDS–PAGE gels and transferred to PVDF membranes. The membranes were then blocked with 5% nonfat milk at room temperature and then probed with specific antibodies (P-STAT3, STAT3, cleaved caspase-3, Bcl-2 and β-actin at a dilution of 1:1000, Cell Signaling Technology, USA) overnight at 4 °C. After incubation with an HRP-conjugated secondary rabbit antibody (1:5000, Cell Signaling Technology, USA), the blots were visualized using Western ECL Blotting Substrates (Bio-Rad, USA).

### Analysis of gene expression by real-time PCR

After 48-hour treatment of 4T1 cells (1 × 10^5^ cells/96-well plate) with 1 µg of WJ-Exo(S3I-201, 301.4 µM), S3I-201 (337.1 µM), 1 µg of WJ-Exo and DMSO, total RNA, including miRNAs, was extracted with Trizol reagent (Gibco, Germany). RNA quantification was performed using NanoDrop (Thermo Fisher Scientific, USA) and 1 µg of total RNA was synthesized using a first-strand cDNA synthesis kit (Takara Biotechnology, Japan).

Quantitative RT-PCR was performed using the Step-One plus instrument (Applied Biosystems, USA) to determine the relative mRNA expression of Bax, Bcl-2, caspase-3 and β2-microglobulin (β2M). Target mRNA expression was evaluated in a 20 µL volume with Syber-green master mix (Ampliqon, Denmark), forward and reverse primers, and cDNA. The primers sequences were: forward primers (Bax: 5’-TGGCAGCTGACATGTTTTCTGAC-3’, Bcl-2: 5’-ATCGCCCTGTGGATGACTGAGT-3’, caspase-3: 5’-GGAAGCGAATCAATGGACTCTGG-3’ and β2M: 5’-ACTGAATTCACCCCCACTGA-3’), reverse primers (Bax: 5’-TCACCCAACCACCCTGGTCTT-3’, Bcl-2: 5’ -GCCAGGAGAAATCAAACAGAGGC-3’, caspase-3: 5’-GCATCGACATCTGTACCAGACC-3’ and β2M: 5’-AAGCAAGCAAGCAGAATTTGGA-3’).

Using β2M as the internal reference, relative gene expression in experimental and control groups was calculated employing the 2^–ΔΔCT^ method, expressing results as fold regulation compared to the control. This analysis was conducted in three independent experiments, each performed in duplicate.

### Examination of cell migration: scratch assay

4T1 cells (7 × 10^5^ cells/well) were seeded in a six-well plate (SPL, South Korea) and allowed to grow to full confluence. Scratches were then made with a 1000-µl pipette tip and cells were washed with PBS, followed by PBS aspiration. The first images were taken at time 0 h. Treatment medium consisting of free S3I-201 (337.1 µM), WJ-Exo(S3I-201) (5 µg exosome loaded with 301.4 µM of S3I-201), WJ-Exo or complete DMEM control medium was then added to the cells. Microscopic images (Zeiss, 4 x magnifications) were taken after 24 h, 48 h and 72 h to ensure consistency of image position. Cell migration was measured using ImageJ software (National Institutes of Health, Bethesda, USA).

### In vivo experiment

Female Balb/c mice aged 6–7 weeks were obtained from the Pasteur Institute in Tehran, Iran. Ethical approval for all animal experiments was obtained from the Institutional Animal Care and Use Committee of Semnan College of Medical Sciences, Iran (Ethic code: IR.SEMUMS.REC.1401.099). Mice were fed a standard laboratory diet and maintained under standardized 12/12-hour light/dark cycle conditions in an air-conditioned room with ambient temperature and humidity.

### Establishment of xenograft tumors

4T1 cells were collected, suspended in PBS and injected into the flanks of female BALB/c mice weighing approximately 20 g at a concentration of 1 × 10^6^ cells. To investigate the antitumor effect in vivo, the mice were randomly divided into four groups of 8 animals each when the tumor volume reached about 100 mm^3^ on day 10. The mice then received intraperitoneal injections of DMSO, S3I-201 (56 µg/dose), WJ-Exo (10 µg of exosome) and WJ-Exo (S3I-201) (10 µg of exosome loaded with 56 µg S3I-201/dose) on days 10, 12 and 14. The tumor volume was measured daily and the volume was calculated using the formula V = π × width × length × height/6. The general health, physical activity and body weight of the mice were monitored daily. The mice were monitored for signs of morbidity throughout the experiment. On day 31, 5 mice in each group were killed by cervical dislocation according to animal ethical guidelines, while 3 mice per group were retained for the assessment of survival rates. Tumor weights were determined and tumor lysates were prepared. The spleens were used after removal for splenocyte proliferation assays and determination of cytokine levels.

### Preparation of tumor cell lysate

For the preparation of tumor cell lysate, the tumor tissue was divided into 1.5 ml microtubes and subjected to five freeze-thaw cycles alternating between liquid nitrogen and a 37 °C water bath. The extracted lysate was then stored at -80 °C for further use.

### Isolation of mouse splenocytes

Mice were euthanized, and during dissection, fur and skin were disinfected with 70% alcohol. Careful manipulation was used to expose the spleen, which was then separated and placed in a 15-ml tube containing 2 ml of ice-cold PBS/FBS. The spleen and PBS/FBS mixture were transferred through a 70-µm cell strainer into a 50-ml tube. After trituration of the spleen without sectioning, 10 ml of PBS/FBS was added and the cell pellet was resuspended in 5 ml of ice-cold ammonium chloride hemolysis buffer. After incubation at 24 °C for 4 min, lysis was stopped with 10 ml ice-cold PBS/FBS and centrifugation at 400×g, 4 °C, for 10 min. The supernatant was discarded and the pellet was resuspended in 2 ml complete RPMI-1640 medium supplemented with 10% FBS and antibiotics. Splenocytes adjusted to a concentration of 1 × 10^6^ cells/ml with viability above 90% (confirmed by trypan blue exclusion) were used for splenocyte proliferation assays and cytokine measurements in the cell culture supernatant.

### Proliferation assay

Antigen-induced lymphocyte proliferation was measured by culturing splenocytes (4 × 10^5^ cells/well) from mice in triplicate on 96-well plates for 48 h at 37 °C/5% CO2. Cultures were treated with tumor lysate (specific stimulator; 2.5 µg/ml) and PHA (non-specific stimulator; 10 µg/ml). A culture plate without stimulation served as a negative control. After further 12-hour incubation, the cultures were harvested and cell proliferation was measured using the MTT assay.

### Measurement of cytokine production

After a period of 60 days following immunization, the animals were euthanized and the spleens were harvested to prepare a single cell suspension. These spleen cells were then cultured on 24-well plates at a density of one million cells/well. Cytokine levels were measured after 72 h in culture supernatants stimulated with PHA 5 µg/ml, tumor lysate at 100 µg/ml or in culture medium alone (negative control). Cytokine concentrations (IFN-γ, TNF-α, IL-4 and IL-1β) in culture supernatants were evaluated using the ELISA method, following the manufacturer’s instructors.

### Statistical analysis

Data, presented as mean ± SD, underwent statistical analysis using One-Way and Two-Way ANOVA followed by Tukey post hoc test for multiple comparisons, along with Kaplan-Meier estimation, in GraphPad Prism 9.4 (GraphPad Software Inc, San Diego, CA, USA). Significance levels were denoted as *P < 0.05, **P < 0.001, ***P < 0.03, ****P < 0.0001 compared to the control group.

## Results

### Characterization MSCs derived from human umbilical cord Wharton’s jelly

**Fig. 1:**
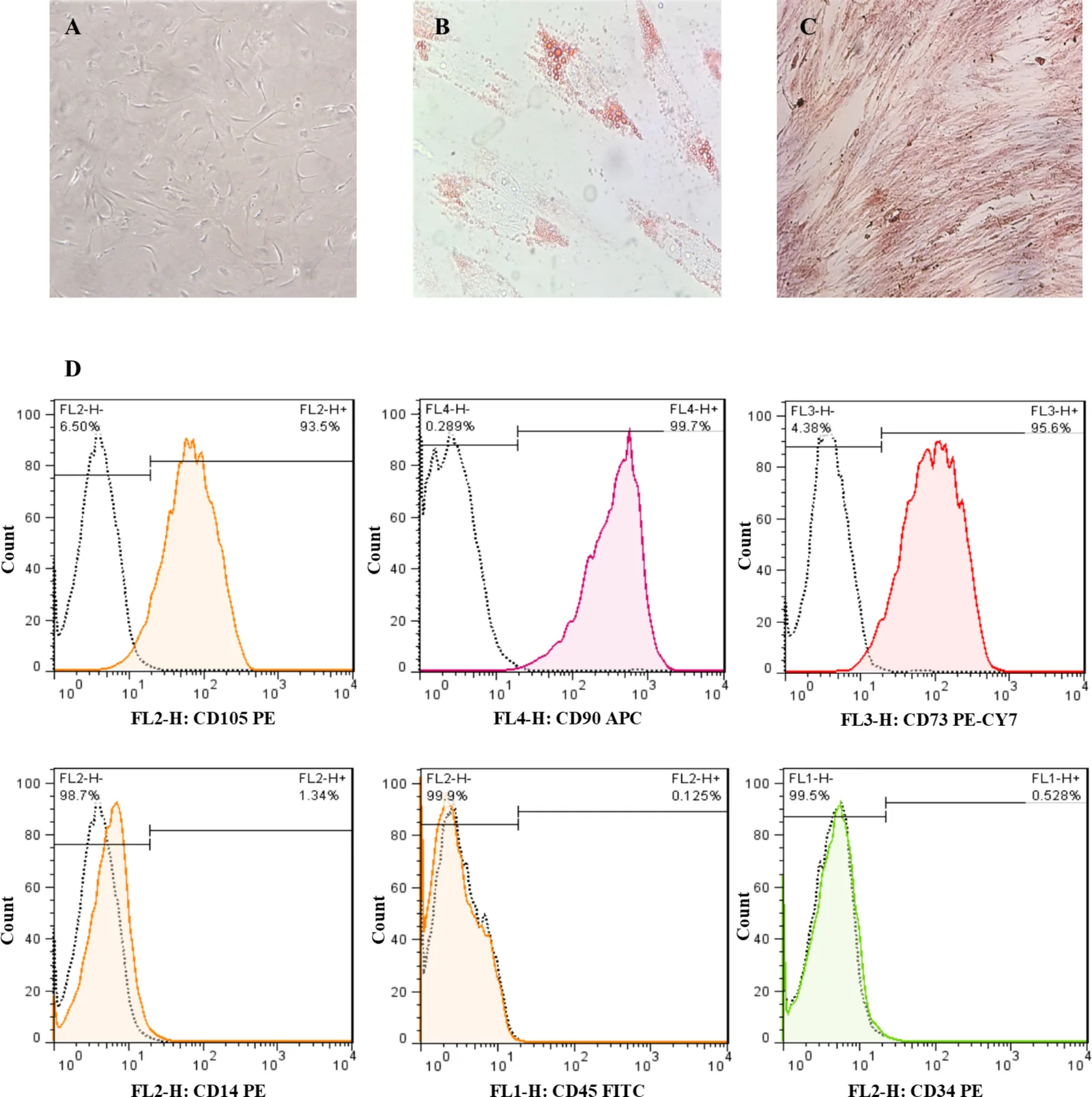
Characterization of WJ-MSCs. (**A**) WJ-MSCs cultures in the third passage show monolayer growth, adherent properties and fibroblast-like morphology under 100x magnification. The white bars represent 50 μm. (**B**) Adipogenic differentiation of WJ-MSCs into adipocytes visualized by staining with Oil Red O for lipid droplets. (**C**) Osteogenic differentiation of WJ-MSCs in osteocytes visualized by Alizarin Red S staining for calcium phosphate accumulation. (**D**) Flow cytometric analysis of surface marker expression of WJ-MSCs with positive markers such as anti-CD105-PE, anti-CD90-APC and anti-CD73-PE-CY7 antibodies and negative markers such as anti-CD14-PE, anti-CD45-FITC and anti-CD34-PE antibodies. The labeled cells were analyzed for marker expression by flow cytometry

Morphological examination of the WJ-MSCs under the inverted light microscope consistently showed a fibroblast-like appearance and adherence features (Fig. 1A). To assess multipotency, specific culture media were used to induce differentiation of adipocytes and osteoblasts, as evidenced by the formation of lipid-filled vesicles and deposition of calcified nodules by oil red O and alizarin red S, respectively (Fig. 1B and C). Flow cytometric analysis also confirmed the presence of MSC-specific markers- CD105 (93.5%), CD90 (99.7%) and CD73 (95.6%) - while the expression of antigens CD14 (1.34%), CD45 (0.125%) and CD34 (0.528%) was minimal (Fig. 1D), confirming cellular homogeneity.

### Characterization of exosomes derived from WJ-MSCs

**Fig. 2:**
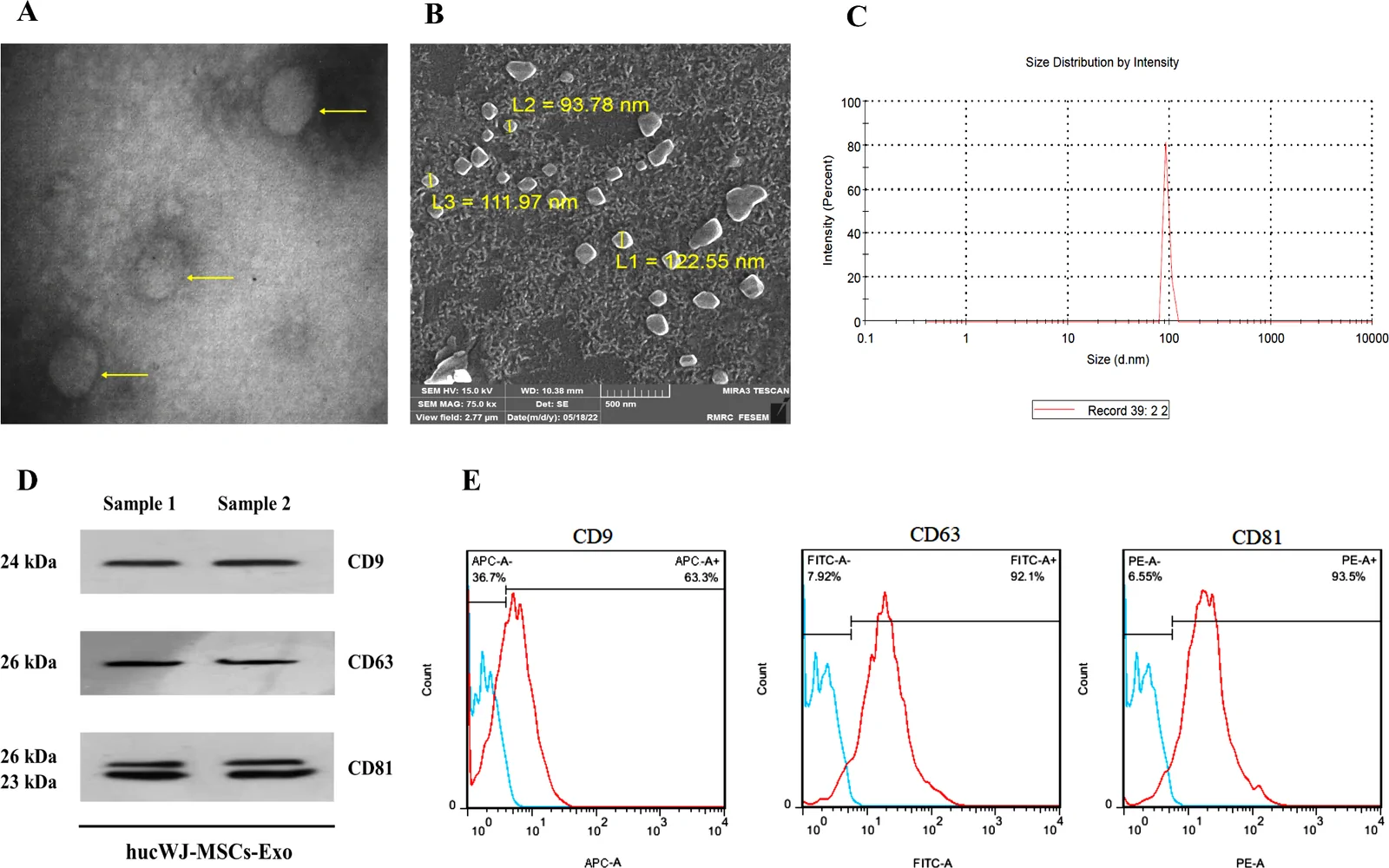
Characterization of WJ-Exo: (**A**) Transmission electron microscopy (TEM) shows exosomes with a characteristic round morphology (yellow arrows), with a scale bar of 200 nm. (**B**) Scanning electron microscope (SEM) image of WJ-Exo, with a scale of 5 μm. (**C**) Size distribution by dynamic light scattering (DLS). (**D**) Western blot analysis of WJ-Exo showing the expression of CD9, CD63 and CD81. Equal amounts of total exosomal proteins were immunoblotted. (**E**) Flow cytometric plots showing the expression of CD9, CD63 and CD81 in WJ-Exo

Exosomes obtained from WJ-MSCs were characterized according to the MISEV2018 guidelines [37], which define exosomes based on morphology, size, and surface markers. Transmission electron microscopy (TEM) and scanning electron microscopy (SEM) showed well-dispersed nanoparticles with complete membranes (Fig. 2A), which exhibited spherical particles with a size of 93.78–122.55 nm in SEM (Fig. 2B). Dynamic light scattering (DLS) revealed an average size of 93.96 ± 5.6 nm (Fig. 2C). Western blot and flow cytometry identified human exosome markers (CD9, CD63 and CD81) with expression levels of 63.3%, 92.1% and 93.5%, respectively (Fig. 2D and E). These results confirm that the purified nanoparticles from WJ-MSCs fulfill the exosome criteria. The amount of exosomes obtained from isolated WJ-MSCs was determined based on the exosomal protein concentration. According to the results of the BCA assay, the protein content of the extracted exosomes was between 0.5 and 1.5 µg/ml culture medium. Overall, the characterization of WJ-MSCs and their derived exosomes was performed in accordance with established guidelines, providing a comprehensive understanding of their morphological and molecular properties.

### S3I-201 was successfully loaded into exosomes using the electroporation

**Fig. 3:**
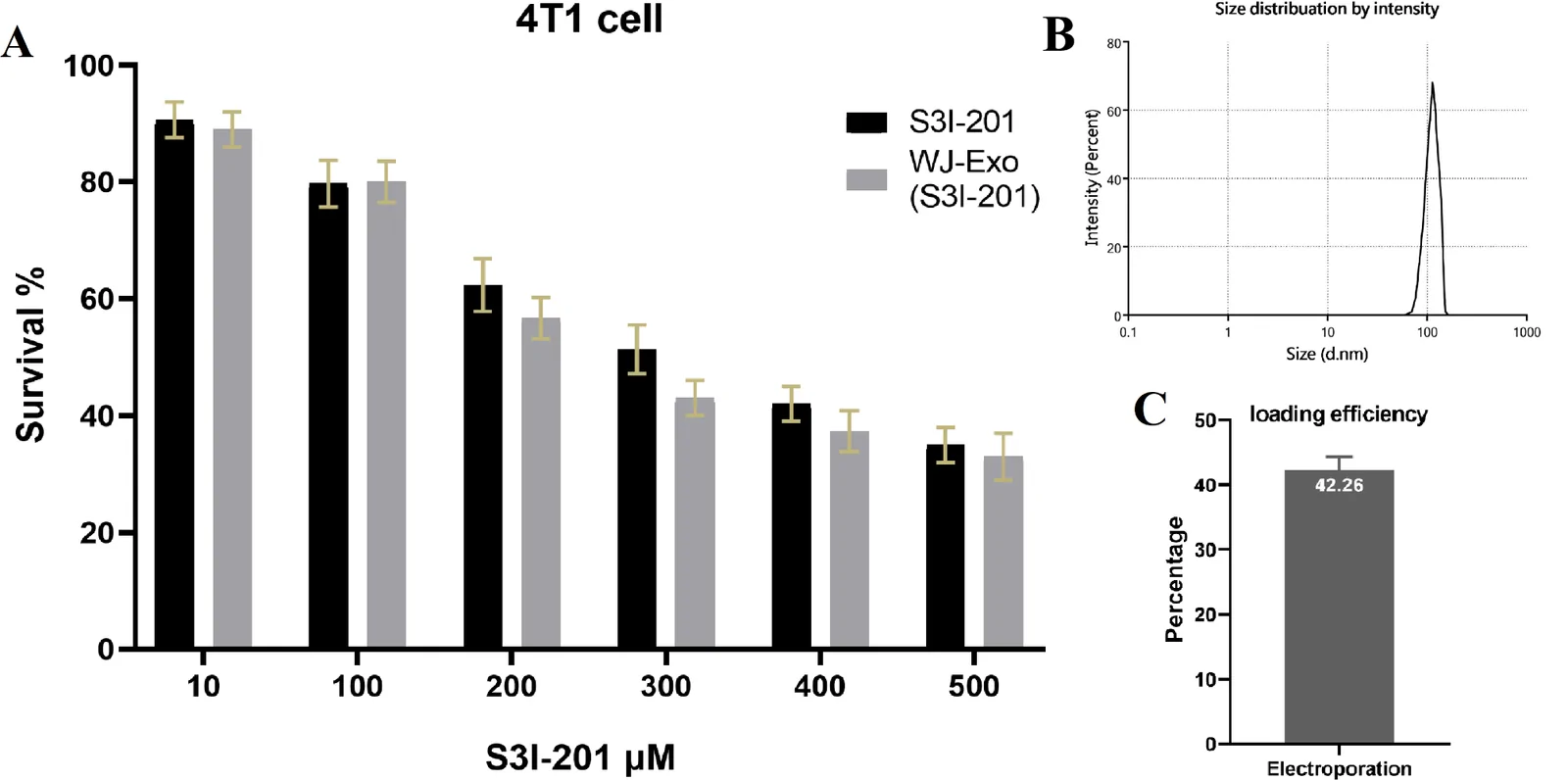
Determination of S3I-201 loading efficiency into exosomes and evaluation of cell viability. (**A**) 4T1 cells were exposed to 1 µg exosome with different concentrations of WJ-Exo (S3I-201) and free S3I-201 (10, 100, 200, 300, 400, 500 µM) for 48 h in a medium-free environment. Cell viability was measured by MTT assay, and WJ-Exo(S3I-201) showed increased cytotoxicity with an IC50 of 301.4 µM, exceeding the cytotoxicity of free S3I-201 with an IC50 of 337.1 µM. (**B**) The dynamic light scattering (DLS) determined size of WJ-Exo after loading was measured to be 117.3 ± 5.6 nm. (**C**) The loading efficiency of S3I-201, calculated from the HPLC area under the curve and the standard curve, was 42.26%

MTT assays were performed before and after loading the exosomes with S3I-201 to determine the IC50 values for the free drug and the nano-formulation, yielding IC50 values of 337.1 and 301.4, respectively (Fig. 3A). DLS was used to evaluate the changes in exosome size after loading with S3I-201. The size of WJ-Exo(S3I-201) (117.3 ± 5.6 nm) increased slightly compared to WJ-Exos (93.96 ± 5.6 nm), possibly due to the integration of S3I-201 into the lipid bilayer and surface adsorption by hydrophobic interaction (Figs. 2C and 3B). These size differences did not hinder entry into 4T1 cells, which is consistent with previous studies indicating increased exosome size after drug loading [38–40]. The HPLC method was used to analyze the loading capacity of the WJ-Exo(S3I-201). The concentration of S3I-201 in the WJ-Exos was determined using the standard curve. The electroporation method achieved an average S3I-201 loading efficiency of 42.26%, which was confirmed by UV-HPLC analysis (Fig. 3C).

### Cytotoxic effects of drug

**Fig. 4:**
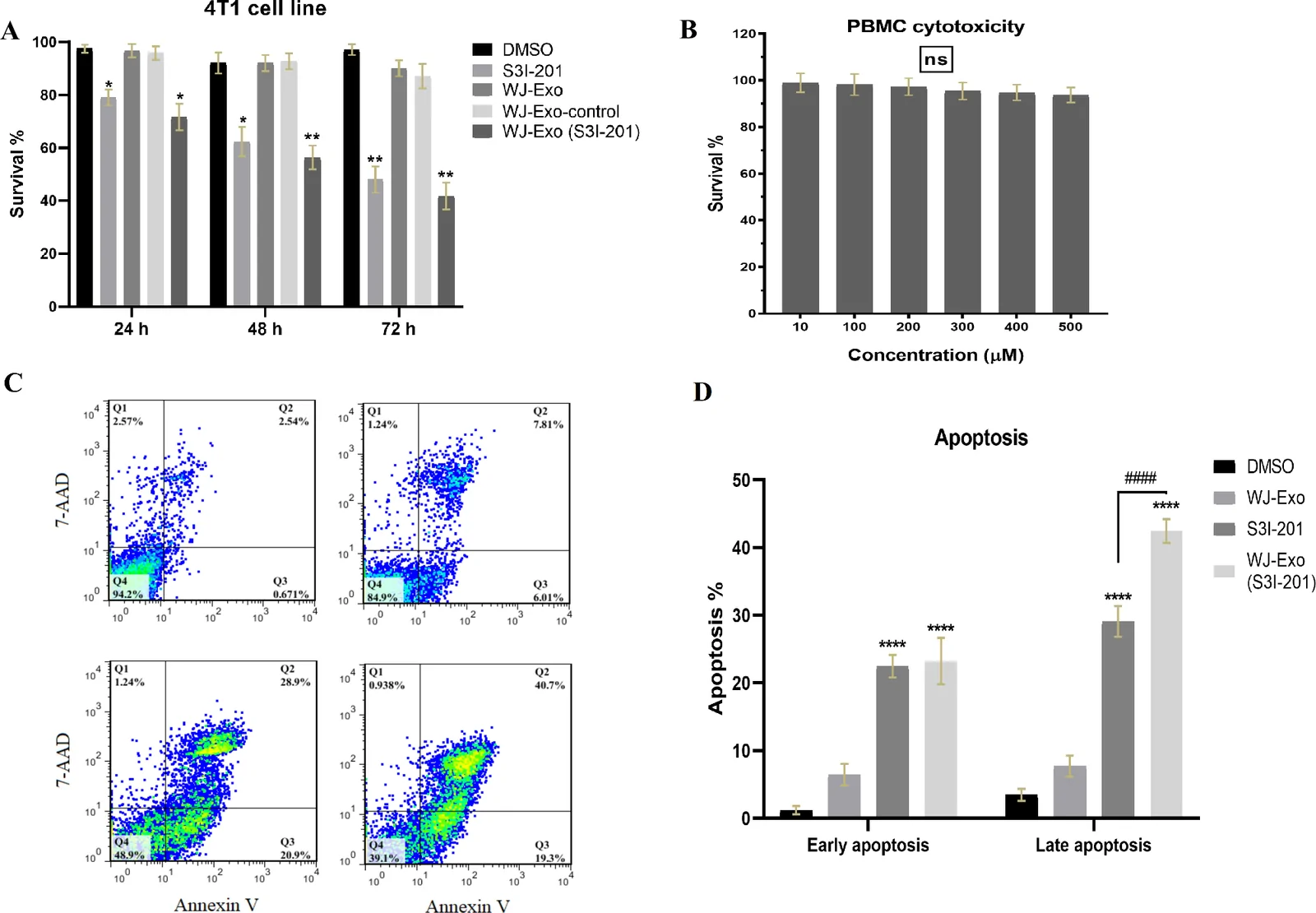
In vitro anti-tumor effects of S3I-201-loaded WJ-Exos. (**A**) Cell viability of 4T1 cells was examined after treatment with DMSO, S3I-201 (IC50: 337.1), WJ-Exo, WJ-Exo control and WJ-Exo(S3I-201, IC50: 301.4 µM) for 24, 48 and 72 h using the MTT assay. WJ-Exo-control means incubation of WJ-Exo with S3I-201 without electroporation to test passive loading of drug to exosomes. (**B**) The viability of healthy cells remained unaffected after 48 h of incubation with different concentrations of free S3I-201. (**C**) 4T1 cells were cultured in a 24-well plate with 5 µg of WJ-Exo (S3I-201) loaded with S3I-201 (IC50: 301.4 µM) and IC50: 337.1 µM of free S3I-201. Flow cytometry was used to evaluate cell apoptosis in each group after 48 h, and (**D**) shows the results of statistical analysis. The data shown are mean ± SD (*n* = 3). (*P < 0.05, **P < 0.001, ***P < 0.03, ****P < 0.0001 vs. the control group) and (# compare between free S3I-201 vs. WJ-Exo(S3I-201)). Statistical significances indicated with * are compared with the DMSO group. Data underwent statistical analysis using One-Way and Two-Way ANOVA followed by Tukey post hoc test for multiple comparisons

We investigated the cytotoxic effects of WJ-Exo(S3I-201) on cancer cell survival by incubating 4T1 cells with DMSO, S3I-201, WJ-Exo, WJ-Exo control and WJ-Exo(S3I-201) for 24, 48 and 72 h, followed by MTT assay for viability. WJ-Exo(S3I-201) exhibited a superior antiproliferative effect on 4T1 cells compared to free S3I-201 (IC50 301.4 µM vs. 337.1 µM) and showed a time-dependent antiproliferative effect compared to S3I-201 alone (Fig. 4A). No significant inhibition was observed in the blank WJ-Exo treated group and the WJ-Exo control, suggesting that WJ-Exo incubation with S3I-201 without electroporation did not affect cancer cell viability. S3I-201 showed no toxic effects on non-cancer cells (Fig. 4B). To further evaluate the pharmacological effect of S3I-201 encapsulated in WJ-Exos, flow cytometry was used to determine the apoptosis of tumor cells induced by treatment with WJ-Exo(S3I-201) (Fig. 4C). Treatment with S3I-201 and WJ-Exo(S3I-201) increased the percentage of apoptotic cells compared to the control group. Moreover, encapsulation of S3I-201 in WJ-Exo significantly increased late apoptosis compared to free S3I-201 (Fig. 4D).

### Effect of the drug on the expression of apoptosis-related genes in 4T1 cells

**Fig. 5:**
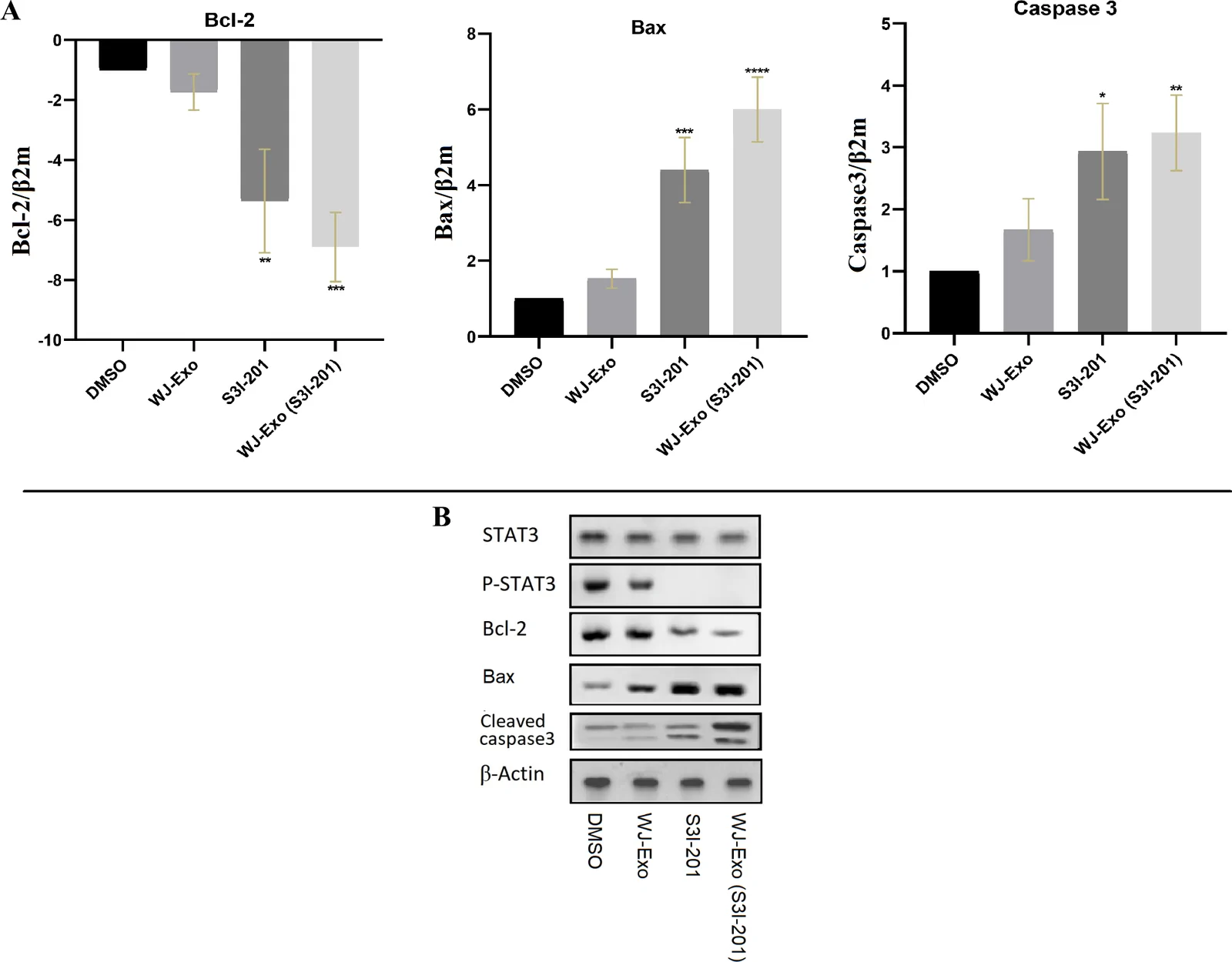
Effects of WJ-Exo(S3I-201) treatment on gene and protein expression in 4T1 cells. (**A**) 4T1 cells (1 × 10^5^ cells/96-well plate) were cultured with 1 µg of WJ-Exo(S3I-201, 301.4 µM), S3I-201 (337.1 µM), 1 µg of WJ-Exo and DMSO for 48-hour. RT-qPCR was used to measure the mRNA expression of Bcl-2, Bax and caspase-3 in 4T1 cells. (*P < 0.05, **P < 0.001, ***P < 0.03, ****P < 0.0001 vs. control group). Statistical significances indicated with * are compared with the DMSO group. (**B**) 4T1 cells (7 × 10^5^ cells/well) were treated with free S3I-201 (337.1 µM), WJ-Exo(S3I-201) (5 µg exosome loaded with 301.4 µM of S3I-201), WJ-Exo and DMSO. Western blot analysis was performed in the study to analyze the expression levels of STAT3, P-STAT3 (Y705), Bcl-2, Bax, and cleaved caspase-3 in 4T1 cells, with β-actin serving as a control for protein loading. Data underwent statistical analysis using One-Way ANOVA followed by Tukey post hoc test for multiple comparisons

To investigate the apoptosis-inducing potential and enhanced therapeutic efficacy of chemotherapeutic agents by drug administration, we examined the expression of apoptosis-related genes (Bcl-2, Bax, and caspase-3) in 4T1 cancer cells treated with DMSO, S3I-201, WJ-Exo(S3I-201), and WJ-Exo. RT–qPCR analyzes showed that WJ-Exo(S3I-201) effectively downregulated the anti-apoptotic gene (Bcl-2) and upregulated the pro-apoptotic genes (Bax and caspase-3). Compared to cells treated with either DMSO or WJ-Exo, the expression of these genes changed significantly in cancer cells incubated with free S3I-201 or WJ-Exo(S3I-201) (Fig. 5A). It is important to note that no significant differences were found between the treatment groups receiving free S3I-201 and those treated with WJ-Exo(S3I-201). These results provide additional evidence of the compound’s ability to promote apoptosis, thereby enhancing the anti-tumor effect of S3I-201.

### WJ-Exo(S3I-201) suppresses STAT3 activation and promotes apoptosis of 4T1 cell

The efficacy of WJ-Exo(S3I-201) in inducing apoptosis and inhibiting STAT3 was further investigated through Western blotting analyses. 4T1 cells were treated with DMSO, S3I-201, WJ-Exo(S3I-201), and WJ-Exo, revealing a noticeable decrease in STAT3 protein expression and phosphorylation at Y705 upon WJ-Exo(S3I-201) treatment. This treatment also correlated with an increased occurrence of cell apoptosis, as demonstrated by heightened expression levels of Bax and Caspase-3, alongside reduced levels of Bcl-2. These findings validate that the inclusion of S3I-201 effectively enhances its cytotoxic efficacy against cancer cells (Fig. 5B).

### WJ-Exo(S3I-201) inhibits 4T1 cell migration

**Fig. 6:**
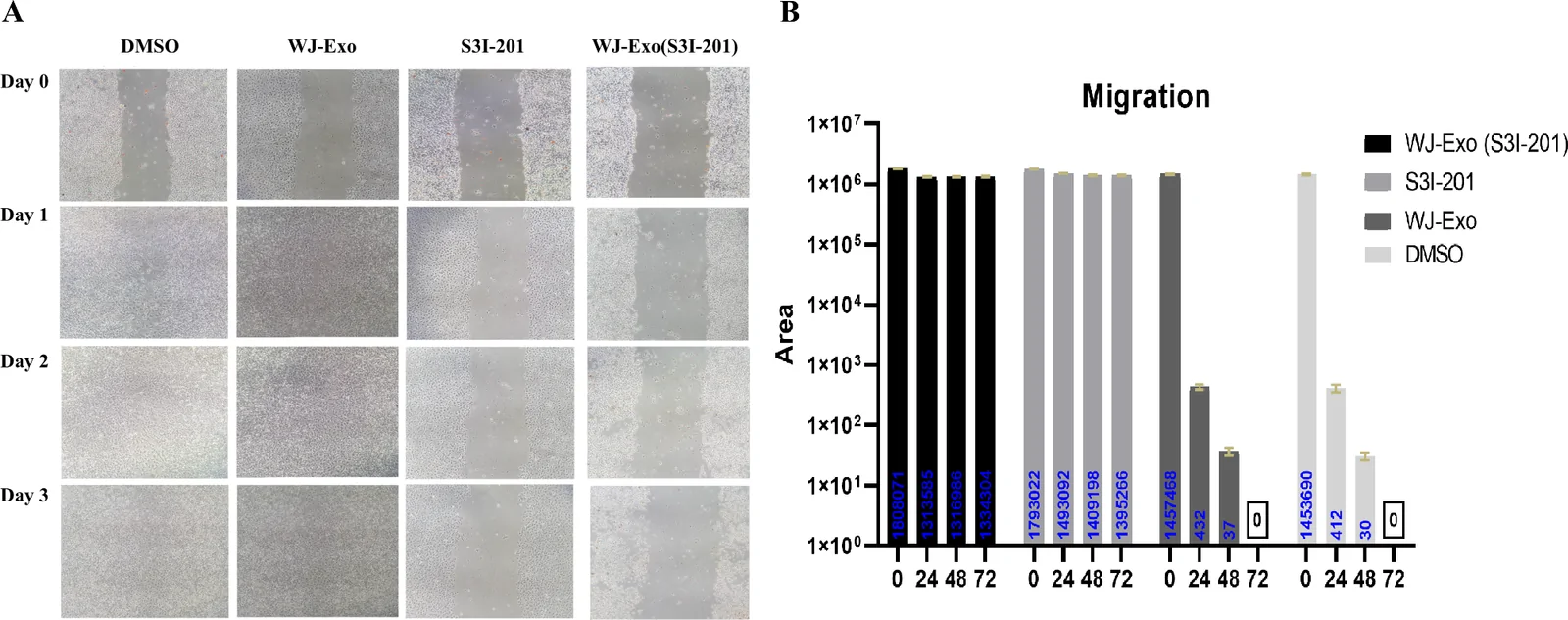
WJ-Exo(S3I-201) inhibits the migration of 4T1 cells. (**A**) A wound healing assay visually shows the initial and final scratch conditions in the 4T1 cell line treated with free S3I-201 (337.1 µM), WJ-Exo(S3I-201) (5 µg exosome loaded with 301.4 µM of S3I-201), WJ-Exo and DMSO. (**B**) Quantitative results for the wound area analysis are presented. (*P < 0.05, **P < 0.001, ***P < 0.03, ****P < 0.0001 vs. the control group). Data underwent statistical analysis using Two-Way ANOVA followed by Tukey post hoc test for multiple comparisons

WJ-Exo(S3I-201) Suppresses 4T1 Cell Migration to evaluate the sustained impact of WJ-Exo(S3I-201)-mediated apoptosis and STAT3 inhibition, we examined its effect on cancer cell migration, a crucial factor in metastasis. Using a wound healing assay in the 4T1 cell line, we observed a significant inhibitory effect of WJ-Exo(S3I-201) and free S3I-201 on cell migration, as evidenced by markedly impaired wound closure compared to DMSO treatment. However, the data showed no significant differences between free S3I-201 and WJ-Exo(S3I-201) (Fig. 6). These findings confirm that WJ-Exo(S3I-201) effectively impedes the migratory capacity of triple-negative breast cancer (TNBC) cells, providing empirical support for its potential therapeutic utility.

### WJ-Exo enhances the in vivo efficacy of S3I-201

**Fig. 7:**
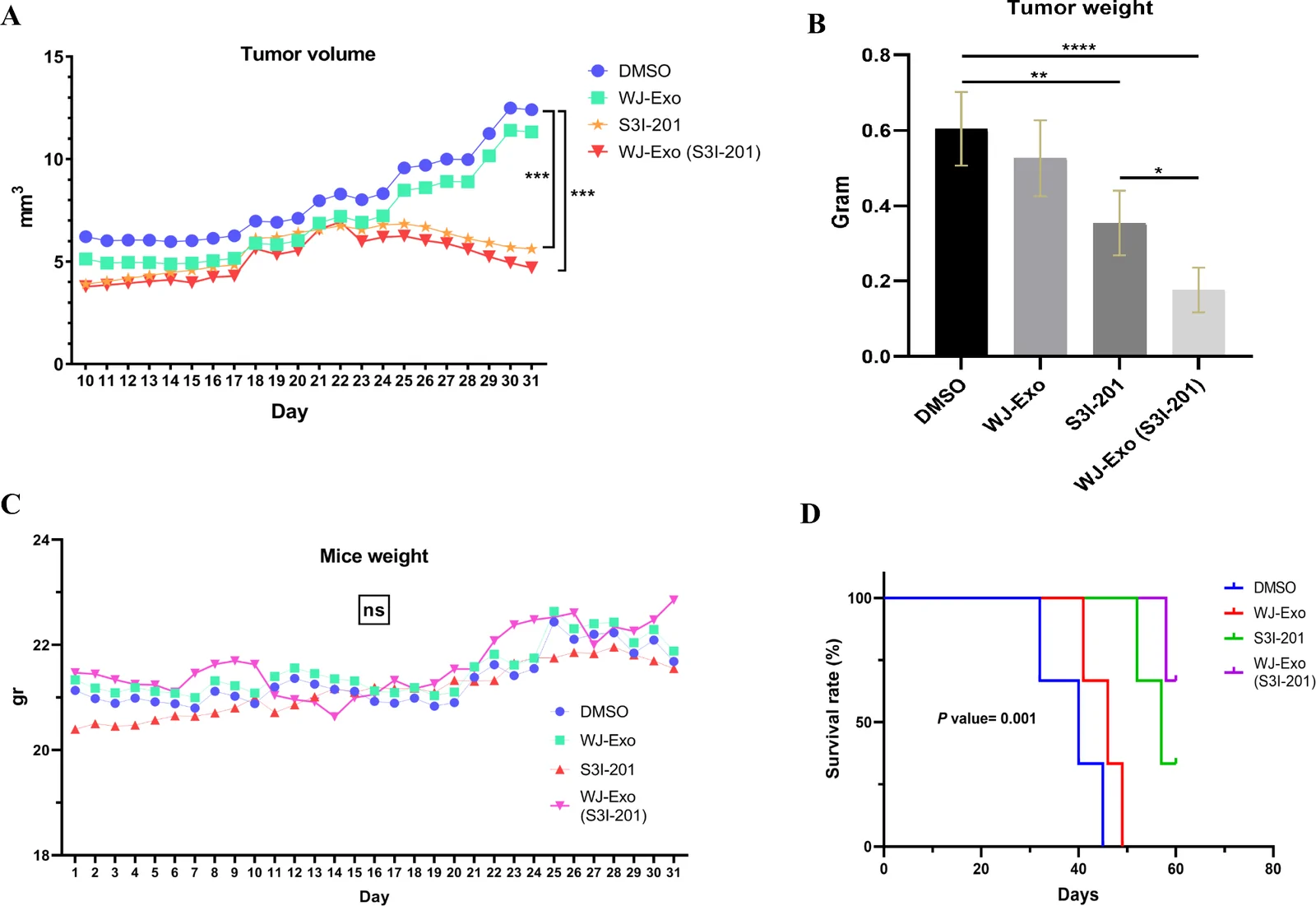
Evaluation of the anti-tumor effects of WJ-Exo, S3I-201 and WJ-Exo(S3I-201) in tumor-bearing mice. (**A**) Determination of tumor volume (*n* = 5/group) and (**B**) excision of tumors for weight measurement after 21 days of treatment. (**C**) Monitoring of daily body weight changes in each group. (**D**) Determination of survival rates (*n* = 3). The mice received intraperitoneal injections of DMSO, S3I-201 (56 µg/dose), WJ-Exo (10 µg of exosome) and WJ-Exo (S3I-201) (10 µg of exosome loaded with 56 µg/dose S3I-201) on days 10, 12 and 14. Values represent mean ± SD (*n* = 8; *P < 0.05, **P < 0.001, ***P < 0.03, ****P < 0.0001 vs. control). Data underwent statistical analysis using One-Way and Two-Way ANOVA followed by Tukey post hoc test for multiple comparisons, along with Kaplan-Meier estimation for survival analysis

Expanding on the aforementioned results, we assessed the in vivo anti-tumor efficacy of WJ-Exo(S3I-201) treatment using a Balb/c mouse model with subcutaneously implanted 4T1 cells, as previously described. Upon reaching a tumor volume of approximately 100 mm^3^, the tumor-bearing mice (*n* = 8 per group) were randomly assigned to four groups and received intraperitoneal administration of therapeutic agents. Subsequent monitoring of anti-tumor activities in 4T1 tumor-bearing mice revealed a significant reduction in tumor volumes (*p* < 0.001) compared to the DMSO group after 21 days of WJ-Exo(S3I-201) and S3I-201 treatment. Remarkably, the WJ-Exo(S3I-201) group exhibited substantial anti-tumor effects (*p* < 0.001) by day 21. However, the data showed no significant differences between S3I-201 and WJ-Exo(S3I-201) groups (Fig. 7A). Furthermore, tumor weight markedly decreased in the WJ-Exo(S3I-201)-treated group compared to free S3I-201 (*p* = 0.024) and DMSO (*p* < 0.0001) groups (Fig. 7B). Importantly, animals in the WJ-Exo(S3I-201) group showed no significant weight loss throughout the study period, indicating minimal acute toxicity (Fig. 7C). The survival rate of mice treated with WJ-Exo(S3I-201) increased compared to the control (*p* value < 0.0003)(Fig. 7D). These consolidated findings affirm the robust anti-tumor efficacy within the WJ-Exo(S3I-201) groups.

**Fig. 8:**
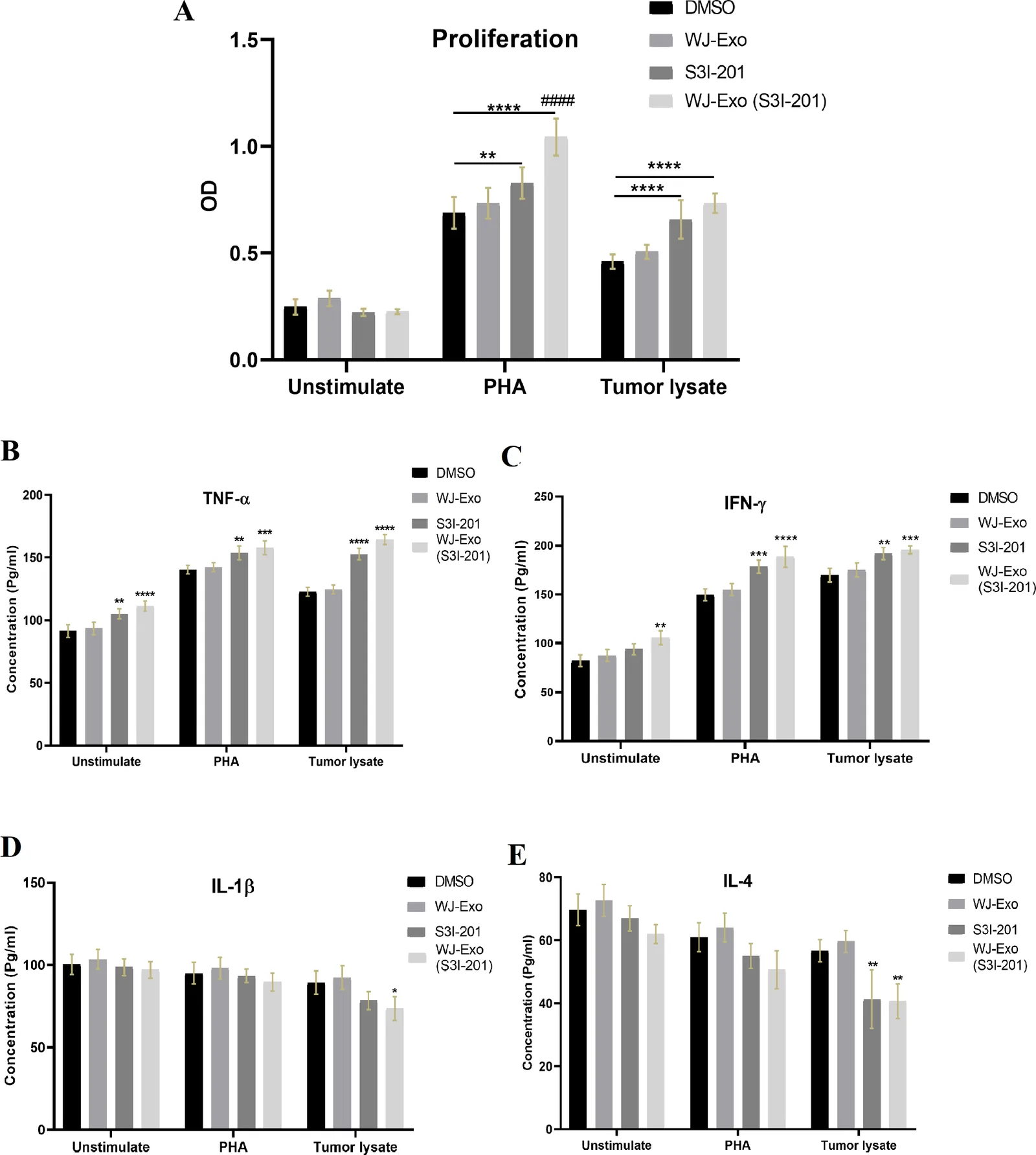
Evaluation of splenocyte proliferation and cytokine levels. (**A**) Splenocyte proliferation: Mouse splenocytes (10^6^ cells/ml) were stimulated with phytohemagglutinin (PHA) and tumor lysate for 72 h at 37 °C and 5% CO2. Non-stimulated splenocytes under identical conditions were used as controls. Statistical significances indicated with * are compared with the DMSO group. Statistical comparisons between the S3I-201 group and WJ-Exo (S3I-201) were labeled with ####. (**B-E**) Cytokine Production: Cytokine levels in cells cultured with PHA and tumor lysate were measured. Murine splenocytes (10^6^ cells/ml) were stimulated for 72 h at 37 °C in 5% CO_2_. Controls comprised non-stimulated splenocytes under the same conditions. Data, representative of at least three (*n* = 3) independent experiments per group, were analyzed via Student’s t-test (*n* = 3; *p < 0.05, **p < 0.001, ***p < 0.03, ***p < 0.0001 vs. control). Data underwent statistical analysis using Two-Way ANOVA followed by Tukey post hoc test for multiple comparisons (**A-E**)

To investigate the impact of WJ-Exo(S3I-201) on tumor cells, we conducted a splenocyte proliferation assay and measured cytokine concentrations. The MTT assay revealed significantly higher splenocyte proliferation in the WJ-Exo(S3I-201)-treated group following tumor lysate stimulation compared to the other groups. Nonspecific PHA stimulation also resulted in a considerable increase in splenocyte proliferation for the WJ-Exo(S3I-201) group compared to the other groups (Fig. 8A). Though, tumor lysate activation could not increase splenocyte proliferation in WJ-Exo(S3I-201) group compared to free S3I-201.

In the assessment of the immunostimulatory properties of WJ-Exo (S3I-201), we measured cytokine concentrations (TNF-α, IFN-γ, IL-4, and IL-1β) in splenocyte supernatant 24 h post WJ-Exo(S3I-201) administration. TNF-α, and IFN-γ concentrations notably increased in the S3I-201 and WJ-Exo(S3I-201) groups following PHA stimulation. The WJ-Exo(S3I-201) group exhibited higher TNF-α and IFN-γ production than the S3I-201 group after PHA stimulation. Furthermore, significantly elevated TNF-α and IFN-γ levels were observed in the S3I-201 and WJ-Exo(S3I-201) groups following tumor lysate stimulation compared to the DMSO group (*p* value < 0.0001), but no significant changes were noted between the DMSO and WJ-Exo groups under tumor lysate and PHA stimulation (Fig. 8B-E).

IL-1β secretion was significantly decreased in the WJ-Exo(S3I-201) group after tumor lysate stimulation, with no significant difference observed between the DMSO and WJ-Exo(S3I-201) groups under PHA incubation (Fig. 8D). Additionally, IL-4 secretion significantly reduced in the WJ-Exo(S3I-201) and S3I-201 groups after tumor lysate stimulation, with no significant difference noted between the DMSO and WJ-Exo(S3I-201) groups under PHA incubation (Fig. 8E). It is important to note that no significant differences were found between the treatment groups receiving free S3I-201 and those treated with WJ-Exo(S3I-201) in all cytokine assessment.

## Discussion

In summary, our approach demonstrates a promising therapeutic strategy for TNBC by targeting the hyper-activated STAT3 protein. Loading WJ-Exo with S3I-201 significantly enhances its anti-tumor effect. The comparison between the two treatment modalities will help in understanding the advantages of utilizing extracellular vesicles as drug delivery vehicles. Furthermore, our findings show that WJ-Exo-based chemotherapy restores Th1-type responses in vivo, thereby enhancing the anti-tumor effect in mice bearing tumors. This research suggests that WJ exo-based chemotherapy, along with free S3I-201, may offer valuable treatment options for TNBC.

In our study, exosomes-loaded with S3I-201 via electroporation showed comparable IC50 values in 4T1 breast cancer cells when compared to free S3I-201. It is noteworthy that our results are in agreement with those of Abas et al. [21] and Munagala et al. [41]. Encapsulation of S3I-201 demonstrated comparable potential in suppressing cell viability, enhancing inhibition of migration, and inducing apoptosis. These results are consistent with other studies in which drugs were loaded into exosomes, such as by Shojaei et al. [42] and Malzer et al. [43].

The results obtained from our MTT assay revealed that the IC50 concentration for the exosome-loaded drug was lower at 301.4 µM compared to 337.1 µM for the free drug. This difference in IC50 values suggests that the exosome-loaded S3I-201 exhibits a more potent anticancer effect compared to the free S3I-201. Hence, the lower IC50 concentration observed for the exosome-loaded drug suggests that encapsulating the drug into exosomes likely enhances its anticancer efficacy, indicating a more potent therapeutic effect compared to the free drug formulation. This disparity in concentrations and the potential benefits of lower doses delivered via exosomes support our hypothesis that the exosome-loaded drug may yield a more robust anticancer response.

In vitro, WJ-Exo(S3I-201) showed increased efficacy in suppressing STAT3, p-STAT3, Bcl-2, Bax and cleaved caspase-3 in 4T1 cells. Furthermore, WJ-Exo (S3I-201) demonstrated superior tumor inhibition in vivo by reducing tumor weight and improving mice survival compared to free S3I-201. This treatment triggered a Th1 cytokine shift characterized by increased IFN-γ and TNF-α levels and decreased IL-4 levels, suggesting a robust anti-tumor response. Notably, WJ-Exo(S3I-201) modulated the imbalance between Th1, Th2 contributing to the recovery of inflammatory status. These results represent a promising therapeutic strategy for TNBC.

In this work, we observed a significant increase in apoptosis of tumor cells and in addition splenocyte proliferation after PHA stimulation by encapsulation of S3I-201 in WJ-Exo. These results are consistent with other studies in which drugs were loaded into exosomes, such as by Shojaei et al. [42] and Malzer et al. [43]. By loading miR-381 mimic into exosomes, Shojaei et al. [42] successfully delivered the drug to TNBCs and promoted their apoptosis in vitro. In addition, the research results of Malzer et al. [43] have shown that the anti-cancer effect of Taxol is significantly enhanced by incorporation into exosomes. They treated different cancer cell types with exosomes loaded with Taxol, and these groups showed the greatest increase in apoptosis compared to the free form of S3I-201. All to gather, these results suggest a specific and more efficient property targeting the tumor.

Furthermore, our data indicated that tumor weight decreased significantly with exosome-loaded S3I-201 compared to free S3I-201, suggesting an improvement in the anti-tumor effect of the drug through encapsulation. Restoration of Th1-type responses in vivo by using WJ-Exo-based chemotherapy further enhances the anti-tumor effect in tumor-bearing mice, which can be explained by the significant decrease in tumor weight. Kalimuthu et al. showed that tumor weight was lower in mice receiving PTX-loaded MSC-derived exosomes than in mice from other groups [44].

To illustrate the cytokine-related results, the increase in IFN-γ and TNF-α secreted by Th1 cells reduces the growth of 4T1 tumors [45]. However, the secretion of IL-4 by Th2 cells exacerbates tumor growth by effectively suppressing the host immune system, thus creating a favorable environment for cancer progression [46]. Therefore, the results of our study suggest that WJ-Exo(S3I-201) has the potential to inhibit breast cancer growth by modulating the Th1/Th2 balance in favor of Th1 dominance. Moreover, recent studies have highlighted the role of STAT3 signaling in the secretion of IL-1β from splenic MDSCs [47, 48]. In addition, the expansion and activity of MDSCs have been linked to inflammatory cytokines such as IL-1β and IL-6. This is orchestrated by the IL-1β-IL-6-STAT3 axis [48]. In the current study, our treatment with WJ-Exo(S3I-201) successfully reduced the secretion of IL-1β. This idea is of great importance in the field of immunotherapy. The activity level of MDSCs has been proposed as a potential indicator of immunotherapy efficacy [49, 50]. It is generally believed that the activity of MDSCs promotes tumor growth and progression. By inhibiting the STAT3 signaling pathway, which plays a crucial role in the expansion of these cells, we can effectively counteract this effect. Consequently, inhibiting the production of IL-1β by MDSCs may represent an important therapeutic strategy to prevent tumor growth. It also promises to minimize treatment-related side effects and improve palliative care [51]. Since immunosuppressive cells in the spleen, such as MDSCs and Treg cells, express the IL-4R [52], a reduction in IL-4 secreted by Th2 leads to decreased activity of these cells, resulting in an enhanced antitumor response.

Therefore, our study has shown that successful inhibition of STAT3 activity not only restrains IL-1β production, but also reveals a potential strategy to control abnormal immune responses. The intricate network of STAT3 interactions underscores its critical role in maintaining immune homeostasis, with evidence suggesting that its dysregulation plays a role in cancer. The study highlights the complexity of STAT3’s immunomodulatory functions and suggests that understanding these interactions could lead to targeted therapeutic interventions to restore balance to the immune system. Overall, this research contributes to the growing body of knowledge in immunology and holds promise for the development of targeted therapies for diseases characterized by dysregulated immune responses.

## Conclusion

In this study, exosomes are used as a sophisticated delivery system for the transport of S3I-201 to 4T1 tumor cells. Administration of WJ-Exo(S3I-201) leads to a noticeable increase in caspase-3 levels and induction of pro-inflammatory Th1 cytokines, accompanied by a corresponding reduction in STAT3 protein levels. These results strongly suggest that the anti-tumor efficacy of WJ-Exo(S3I-201) is associated with the dual modulation of STAT3 suppression and the coordinated release of pro-inflammatory cytokines. The use of WJ-Exo(S3I-201) compared to S3I-201 not only effectively suppresses STAT3, but also enhances the anti-tumor effects of S3I-201. Furthermore, WJ-Exo-based chemotherapeutic interventions have been shown to restore Th1-type responses in vivo, synergistically enhancing the anti-tumor effects observed in mice with tumors. This versatile and advanced therapeutic approach is promising for the further development of cancer therapeutics, offering a tailored and potent option for addressing the complexities of TNBC.

## Data Availability

No datasets were generated or analysed during the current study.
